# Maternal Fructose Intake Induces Insulin Resistance and Oxidative Stress in Male, but Not Female, Offspring

**DOI:** 10.1155/2015/158091

**Published:** 2015-02-11

**Authors:** Lourdes Rodríguez, Paola Otero, María I. Panadero, Silvia Rodrigo, Juan J. Álvarez-Millán, Carlos Bocos

**Affiliations:** ^1^Facultad de Farmacia, Universidad CEU San Pablo, Urbanización Montepríncipe, Boadilla del Monte, 28668 Madrid, Spain; ^2^CQS Laboratory, C/Artistas 1, 28020 Madrid, Spain

## Abstract

*Objective*. Fructose intake from added sugars correlates with the epidemic rise in metabolic syndrome and cardiovascular diseases. However, consumption of beverages containing fructose is allowed during gestation. Recently, we found that an intake of fructose (10% wt/vol) throughout gestation produces an impaired fetal leptin signalling. Therefore, we have investigated whether maternal fructose intake produces subsequent changes in their progeny. *Methods*. Blood samples from fed and 24 h fasted female and male 90-day-old rats born from fructose-fed, glucose-fed, or control mothers were used. *Results*. After fasting, HOMA-IR and ISI (estimates of insulin sensitivity) were worse in male descendents from fructose-fed mothers in comparison to the other two groups, and these findings were also accompanied by a higher leptinemia. Interestingly, plasma AOPP and uricemia (oxidative stress markers) were augmented in male rats from fructose-fed mothers compared to the animals from control or glucose-fed mothers. In contrast, female rats did not show any differences in leptinemia between the three groups. Further, insulin sensitivity was significantly improved in fasted female rats from carbohydrate-fed mothers. In addition, plasma AOPP levels tended to be diminished in female rats from carbohydrate-fed mothers. *Conclusion*. Maternal fructose intake induces insulin resistance, hyperleptinemia, and plasma oxidative stress in male, but not female, progeny.

## 1. Introduction

In the last few decades, obesity, metabolic syndrome, and diabetes have escalated to epidemic proportions in many countries worldwide. These metabolic diseases are multifactorial resulting from genetic, physiological, behavioural, and environmental influences. Genetic influence alone does not suffice to explain the rate at which these diseases have increased [1]. In fact, several studies demonstrate that metabolic events during pre- and postnatal development modulate metabolic disease risks in later life [2]. Among them, feeding conditions likely constitute one of the most influential parameters on the health of the adult [3]. Thus, diet manipulation in mothers during critical developmental periods (such as gestation and/or the early postnatal) has been used to identify their contribution to obesity and diabetes development in offspring [4]. Given the current worldwide shift toward a westernized lifestyle, there is an urgent need to address the relationship between the quality and quantity of nutrient intake during pregnancy and/or lactation and the metabolic fate of the offspring [5].

Fructose, present in added sugars such as sucrose and high-fructose corn syrup, has been linked to obesity and metabolic syndrome [6–8]. Experimental studies have shown that fructose can induce leptin resistance and features of metabolic syndrome in rats, whereas glucose intake does not [9–11]. Clinical studies also support fructose as a cause of metabolic syndrome [12–14].

Interestingly, sex-dependent differences in the influence of fructose for inducing metabolic diseases have been reported. Thus, women, but not men, exhibit an association between fructose consumption and an increased risk of type 2 diabetes mellitus. In relation to this, it has been shown that female rats subjected to fructose have a more detrimental response than their male counterparts. Fructose-fed male rats were resistant to the hepatic effects of leptin, whereas fructose-fed females had no signs of leptin resistance but had hyperinsulinemia and altered glucose tolerance test [15]. In contrast, in high-fructose-fed rodents, oxidative stress was observed in male, but not in female, rats [16].

Since it has been well-established that fructose intake modifies lipidemia in laboratory animals and humans [11, 17] and maternal diet manipulations can affect the progeny, it might be speculated that fructose administration during gestation and/or lactation could cause metabolic changes in the offspring. Unfortunately, studies investigating altered maternal nutrition have used quite different experimental designs to determine the role of fructose [18–24].

In our previous report, we investigated the effects of a low fructose intake throughout gestation in mothers and their fetuses [25], and we obtained intriguing results. Fructose-fed mothers presented a diminished leptin response to fasting and refeeding, their fetuses displaying an impaired transduction of the leptin signal, and these findings were not observed in glucose-fed rats. Therefore, the present study was designed to determine whether an intake of liquid fructose (10% wt/vol) during pregnancy had long-term consequences on the offspring. Furthermore, special attention has been given to determine whether any potential consequence of the treatment differed between female and male offspring.

## 2. Material and Methods

### 2.1. Animals and Experimental Design

Female Sprague-Dawley rats weighing 200–240 g were fed* ad libitum* a standard rat chow diet (B&K Universal, Barcelona, Spain) and housed under controlled light and temperature conditions (12 h light-dark cycle; 22 ± 1°C). The experimental protocol was approved by the Animal Research Committee of the University CEU San Pablo, Madrid, Spain. The experimental protocol to which pregnant rats were subjected was the same as previously reported [25]. Briefly, pregnant animals were randomly separated into a control group, a fructose-supplemented group (fructose), and a glucose-supplemented group (glucose). Fructose and glucose were supplied as a 10% (wt/vol) solution in drinking water throughout gestation. Control animals received no supplementary sugar. Pregnant rats were allowed to deliver and, on the day of birth, each suckling litter was reduced to nine pups per mother. After delivery, both mothers and their pups were maintained with water and food* ad libitum*. It is remarkable that these animals (mothers and pups) received no subsequent additive in the drinking water. On the 21st day after delivery, the lactating mothers were removed to stop the suckling period and pups were separated by gender. When the progeny were 90 days old, they were subjected to 24-hour fasting. After drawing a basal blood sample from the tail vein, pellets were removed from the cages. After 24 h fasting, a second blood sample from the tail vein was obtained. Blood samples were collected into EDTA (1 mg/mL) tubes and placed on ice. Samples were then centrifuged, and plasma was stored at −80°C until processed for glucose, insulin, leptin, and other determinations. Where two or three pups from one litter were used, their data were averaged.

### 2.2. Determinations

Plasma aliquots were used to measure glucose by an enzymatic colorimetric test (Spinreact, Girona, Spain). NEFA (nonesterified (“free”) fatty acids) (Wako, Neuss, Germany), glycerol (Sigma Chemical, St. Louis, MO), triglycerides, and uric acid (Spinreact) were measured using commercial kits. Ketone bodies were measured using a kinetic method (Randox Laboratories, United Kingdom). Insulin was determined in plasma samples using a specific ELISA kit for rats (Mercodia, Uppsala, Sweden). Leptin and adiponectin were assayed in plasma samples using a specific enzyme immunoassay (EIA) kit for rats (Biovendor, Brno, Czech Republic, and Millipore, Bedford, MA, resp.).

Estimates of insulin resistance were calculated as previously described [15, 25] by determination of the following indexes from the 24 h fasting plasma glucose and insulin values: homeostasis model assessment of insulin resistance (HOMA-IR) and insulin sensitivity index (ISI). The HOMA-IR was calculated as the product of the fasting plasma glucose (FPG) and insulin (FPI) divided by a constant, 22.5. FPI was expressed in microunits per milliliter and FPG as millimoles per liter [26]. The ISI was calculated as the ratio 2/[(FPI × FPG) + 1], expressing FPI and FPG in micromoles per liter [15]. Additionally, estimates of insulin sensitivity were also calculated in fed-state as previously described [25] by determination of the ratio between glucose levels (in milligrams per deciliter) and insulin values (in microunits per milliliter).

Finally, plasma aliquots were also used to determine the oxidative stress state. The concentration of malondialdehyde (MDA) in plasma was measured as a marker of lipid peroxidation using the method previously described [27], by measuring the fluorescence of MDA-thiobarbituric acid (TBA) complexes at 515 nm/553 nm excitation/emission wavelengths. Further, the advanced oxidation protein products (AOPP) in plasma were determined as a protein oxidative stress biomarker using the spectrophotometric technique previously described [28, 29]. The AOPP concentrations were expressed as *μ*mol/L of chloramine-T equivalents.

### 2.3. Statistical Analysis

Results were expressed as mean ± SE. Treatment effects were analyzed by one-way ANOVA. When treatment effects were significantly different (*P* < 0.05), means were tested by the Tukey multiple range test, using a computer program SSPS (version 15). When the variance was not homogeneous, a post hoc Tamhane test was performed.

## 3. Results

### 3.1. Ingestion of a 10% wt/vol Fructose Solution throughout Gestation Affects Leptinemia in Male Progeny

As shown in Table 1, neither fructose nor glucose intake throughout pregnancy produced alterations in the body weight of the male and female progeny.

Plasma NEFA, glycerol, and triglycerides concentrations were similar in the male rats from carbohydrate-fed mothers with respect to control values (Table 1). Male rats from carbohydrate-fed mothers showed higher levels of plasma adiponectin levels. Although glycemia and insulinemia showed no differences between the three groups (Table 1), glucose/insulin ratio tended to be lower in the male animals from fructose-fed mothers compared to the animals from control and glucose-supplemented rats (Table 1). Interestingly, male descendents from fructose-supplemented rats turned out to be clearly hyperleptinemic (Table 1).

On the other hand, since insulinemia and glycemia were similar in the female rats from carbohydrate-fed mothers with respect to control values, glucose/insulin ratio was found to be similar for the female progeny of control, fructose-fed, and glucose-fed pregnant rats (Table 1). Similar findings were recorded for plasma adiponectin levels. However, plasma NEFA, glycerol, and triglycerides concentrations were lower in female animals from glucose-fed pregnant rats in comparison to the other two groups (Table 1). In contrast to males, female descendents from fructose-supplemented rats showed similar values in their leptinemia to the animals from control and glucose-supplemented mothers (Table 1).

### 3.2. Ingestion of a 10% wt/vol Fructose Solution throughout Gestation Affects Insulin Sensitivity in Fasted Male Progeny

To confirm a possible disturbance in glucose homeostasis in male, but not in female, rats born of mothers supplemented throughout pregnancy with liquid fructose, we subjected the progeny of the three experimental groups to 24 h fasting and measured plasma parameters related to insulin resistance.

**Figure 1:**
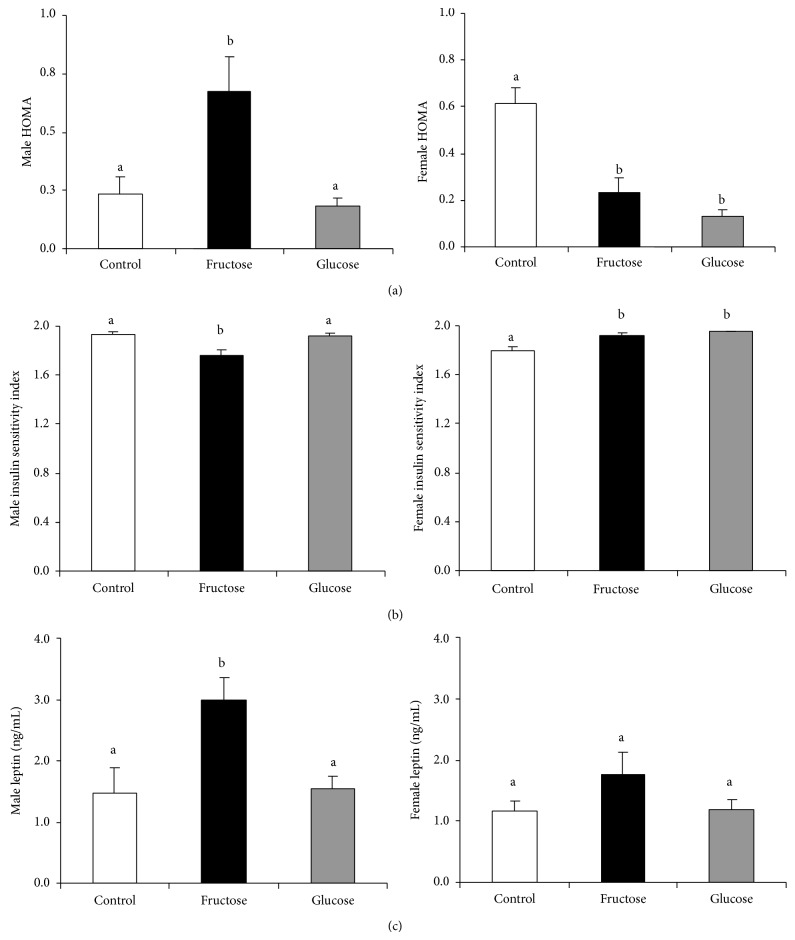
Fructose in pregnancy produces insulin resistance and hyperleptinemia in progeny. Male and female (a) HOMA-IR and (b) ISI ratios and (c) leptinemia of 24-hour fasted 90-day-old progeny from control, fructose-fed, and glucose-fed pregnant rats. Data are means ± SE; *n* = 10–12 animals from four litters. Different letters indicate significant differences between the groups (*P* < 0.05).

After 24-hour fasting, plasma glucose, NEFA, glycerol, ketone bodies, and triglycerides concentrations did not show any differences in the male rats from carbohydrate-fed mothers with respect to control values (Table 2). Similar findings were recorded for plasma adiponectin levels. However, insulinemia was significantly higher in male animals from fructose-fed pregnant rats in comparison to the other two groups (Table 2). Thus, HOMA-IR and ISI ratios were significantly different in the male animals from fructose-fed mothers compared to the male animals from control and glucose-supplemented rats (Figures 1(a)-1(b)), and, therefore, these findings confirmed that an insulin resistant state exists in male progeny of fructose-fed mothers. In accordance with this result, male descendents from fructose-supplemented rats showed a clear increase in their plasma leptin concentrations (Figure 1(c)).

After 24-hour fasting, plasma glucose, NEFA, glycerol, ketone bodies, adiponectin, and triglycerides levels were similar in the female rats from carbohydrate-fed mothers with respect to the control values (Table 2). However, insulinemia was lower in female animals from carbohydrate-fed pregnant rats in comparison to the control group (Table 2). Thus, HOMA-IR and ISI ratios were significantly different in the female animals from carbohydrate-supplemented mothers compared to the female animals from control rats (Figures 1(a)-1(b)), these being more pronounced in the group from the glucose-fed mothers. Thus, not only did female rats from fructose-fed mothers not show an insulin resistant state as seen in male progeny, but female rats from carbohydrate-fed mothers showed improved insulin sensitivity. In fasted female descendents from fructose-supplemented rats, although plasma leptin concentrations tended to be higher than in the other two groups, the effect was not significant (Figure 1(c)).

### 3.3. Ingestion of a 10% wt/vol Fructose Solution throughout Gestation Affects Plasma Oxidative Stress in Male Progeny

**Figure 2:**
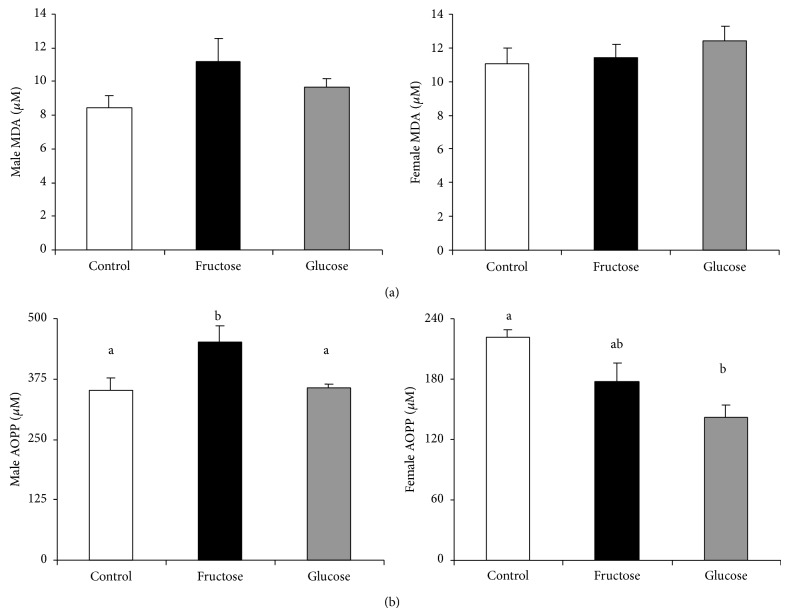
Fructose in pregnancy influences plasma oxidative stress in progeny. Male and female (a) plasma MDA (b) and AOPP values of 90-day-old progeny from control, fructose-fed, and glucose-fed pregnant rats. Data are means ± SE; *n* = 10–12 animals from four litters. Different letters indicate significant differences between the groups (*P* < 0.05).

Since fructose intake during pregnancy seems to produce insulin resistance and hyperleptinemia in the male progeny, parameters which have been related to metabolic syndrome, we also checked whether other features related to that disturbance could be affected. Thus, as shown in Figure 2, plasma MDA and AOPP, which would indicate lipid and protein oxidation, respectively, tended to be augmented in the male rats from fructose-fed mothers in comparison to the animals from control and glucose-fed pregnant rats, becoming significant for AOPP levels. In contrast, plasma AOPP, but not MDA, were diminished in the female progeny of carbohydrate-fed mothers (Figure 2), this effect being significantly different in the progeny from glucose-fed mothers.

Interestingly, male rats from fructose-supplemented mothers showed higher uricemia (3.4 ± 0.5; 5.4 ± 0.3; and 3.4 ± 0.4 mg/dL, for males born from control, fructose-fed, and glucose-fed mothers, resp., *P* < 0.05). In comparison, females from carbohydrate-fed mothers showed lower levels of plasma uric acid than the control group, this effect being significantly different in the progeny from glucose-fed mothers (4.1 ± 0.3; 3.4 ± 0.3; and 2.3 ± 0.2 mg/dL, for female progeny from control, fructose-fed, and glucose-fed mothers, resp., *P* < 0.05).

## 4. Discussion

In our previous study [25], we reported an impaired hepatic transduction of the leptin signal in the fetuses from fructose-fed, but not glucose-fed, pregnant rats and related it to a diminished leptin response to fasting and refeeding observed in their mothers. Since it has been proposed that a period of relative hypoleptinemia (or, in our case, leptin resistance) during development may induce some metabolic adaptations that underlie developmental programming [30], we speculated whether the findings found in our previous study [25] could be responsible for a developmental programming of those progeny and produce some metabolic disturbances, when adult.

Thus, male progeny born of fructose-fed rats presented higher leptinemia versus the other two groups, and that hyperleptinemia was observed in both fed and fasting conditions. In accordance with this finding, fasting insulinemia was higher in the male progeny from fructose-fed mothers, all these results being consistent with the insulin resistant state found in these animals (measured as HOMA and ISI ratios). The presence of hyperinsulinemia in fasted male fructose-fed progeny along with hyperleptinemia could also indicate leptin resistance at the level of pancreatic islets [31]. Since insulin stimulates adipogenesis and leptin production in adipocytes whereas leptin inhibits the production of insulin in pancreatic *β*-cells, a prolonged elevation of plasma leptin levels would result in dysregulation of the adipoinsular axis and a corresponding failure to suppress insulin secretion [32]. In fact, in the present study, although the male progeny from fructose-fed mothers was hyperleptinemic in both fed and fasted conditions, fasting produced a lower impact on insulin levels in comparison to the other two groups (12.1-, 4.3-, and 15.2-fold reduction, for control, fructose, and glucose groups, resp.). In relation to this, it has been reported that adipocytes from male offspring of lactating mothers consuming fructose spontaneously released more leptin than control rat-derived adipocytes and also displayed impaired response to insulin stimulation [4]. Since the progeny from fructose-fed mothers were already leptin resistant when they were fetuses [25], the male progeny of fructose-fed pregnant rats could present a vicious circle (leptin resistance, hypersecretion of insulin, and increasing insulin resistance) [23, 33].

On the other hand, it has been demonstrated that fructose is much more reactive than glucose with respect to participation in glycosylation reactions, which represent an important source of free radicals. In accordance with this fact, rats fed fructose (25% wt/vol) exhibited a significant increase in lipid peroxidation products, compared with rats fed the same amount of glucose or sucrose and those given pure water [34]. Moreover, it has been reported that a short-term administration of a fructose-rich diet to normal rats promotes an increase in several glycoxidative stress markers [16, 35]. Further, as a reducing sugar, fructose reacts with protein molecules to form toxic advanced glycation end-products (AGEs) and also induces protein oxidation possibly through the formation of hydroxyl radicals [36]. Both AGEs and AOPP appear to be involved in the pathogenesis of inflammation, diabetes complications, and cardiovascular diseases. In the present work, the male progeny from fructose-fed dams showed increased plasma levels of protein oxidation products, measured as AOPP. It is worthwhile to mention that AOPP levels have been associated with metabolic syndrome, since high levels of AOPP have been positively correlated with insulin and HOMA levels [37]. Given that it has been proposed that AGEs could accumulate indefinitely on long-lived molecules such as collagen and DNA [38], it might be speculated that fructose intake during pregnancy could promote oxidation products formation in fetuses and these toxic compounds would accumulate, later appearing in the plasma of progeny.

Related to that increase in plasma oxidative stress of progeny from fructose-fed rats, we also determined uricemia and, surprisingly, male rats born to fructose-fed mothers turned out to be hyperuricemic. Some authors consider hyperuricemia in metabolic syndrome to be the consequence of elevated serum insulin levels, which have been shown to stimulate renal reabsorption of uric acid. In fact, thiazolidinediones which improve insulin sensitivity and lower insulin levels reduce the level of serum uric acid in diabetic patients. Conversely, Nakagawa et al. (2006) demonstrated that the reduction of uric acid levels with a xanthine oxidase inhibitor improved insulin sensitivity. Accordingly, in the present study, fructose-fed male progeny presented hyperuricemia along with insulin resistance. Interestingly, the xanthine oxidase pathway, which is related to uric acid production, has been shown to generate oxidants [39]. Our findings would agree with this result since the hyperuricemic male progeny also presents an increase in plasma oxidative stress. In fact, a recent study has shown a close relationship between fructose, hyperuricemia, oxidative stress, and diabetes [40].

The most prominent result found here is that the intake of just a small amount of fructose (10%) throughout gestation produces a clear impairment in the insulin action, hyperleptinemia, and other features of metabolic syndrome such as high values of oxidative stress biomarkers and uricemia in male progeny. However, female progeny born of fructose-fed mothers did not show any of these characteristics of metabolic syndrome. Nevertheless, as has been described, females born to mothers subjected to undernutrition expressed a programmed phenotype only in the presence of a high-fat diet, whereas the male progeny could manifest it independently of postnatal nutrition [30]. Therefore, it is possible that postnatal hypercaloric nutrition could amplify all these metabolic abnormalities induced by the fructose-fed fetal programming, and this deserves further investigation.

Finally, since the carbohydrate was administered only during gestation, we can assume that the effects of fructose intake would occur during intrauterine development. As previously reported [25], fetuses from fructose-fed mothers presented leptin resistance. Leptin has been implicated in the regulation of insulin secretion by islets and, in fact, leptin may influence the normal proliferation of pancreatic *β*-cells which occurs in the neonatal period [30]. If it is assumed that an interrelated endocrine insulin-leptin feedback system exists, we could speculate that the adipoinsular axis has been affected by fructose intake throughout pregnancy.

## 5. Conclusion

Maternal fructose intake seems to provoke features of metabolic syndrome, namely, insulin resistance, hyperleptinemia, hyperuricemia, and plasma oxidative stress in male, but not female, progeny.

## Figures and Tables

**Table 1:** Body weight and plasma analytes in fed 90-day-old progeny from fructose- or glucose-supplemented mothers.

	Male		
	Control	Fructose	Glucose
Body weight (g)	423.7 ± 12.3	403.4 ± 12.8	384.7 ± 21.3
Glucose (mg/dL)	133.4 ± 4.3	128.7 ± 3.2	138.0 ± 1.0
Insulin (*μ*g/L)	0.53 ± 0.05	0.62 ± 0.06	0.47 ± 0.08
Glucose/insulin ratio	10.4 ± 0.4	8.1 ± 0.9	11.9 ± 1.4
Triglycerides (mg/dL)	95.6 ± 8.0	99.9 ± 2.2	82.5 ± 5.7
NEFA (mM)	0.45 ± 0.10	0.56 ± 0.15	0.46 ± 0.07
Glycerol (mg/dL)	2.87 ± 0.29	3.18 ± 0.22	2.55 ± 0.18
Adiponectin (*μ*g/mL)	18.9 ± 1.1^a^	24.3 ± 0.9^b^	25.1 ± 1.8^b^
Leptin (ng/mL)	6.35 ± 0.57^a^	11.20 ± 1.44^b^	7.08 ± 0.92^a^

	Female		
	Control	Fructose	Glucose

Body weight (g)	260.4 ± 9.6	258.7 ± 6.4	253.7 ± 4.2
Glucose (mg/dL)	139.3 ± 7.0	134.1 ± 2.8	142.7 ± 1.8
Insulin (*μ*g/L)	0.54 ± 0.05	0.49 ± 0.07	0.40 ± 0.05
Glucose/insulin ratio	10.7 ± 1.9	10.0 ± 0.7	14.7 ± 1.0
Triglycerides (mg/dL)	57.2 ± 2.9^a^	55.9 ± 8.1^a^	32.0 ± 4.3^b^
NEFA (mM)	0.48 ± 0.09	0.50 ± 0.09	0.37 ± 0.04
Glycerol (mg/dL)	2.81 ± 0.21	2.68 ± 0.29	2.10 ± 0.14
Adiponectin (*μ*g/mL)	43.9 ± 4.0	48.7 ± 3.5	46.7 ± 3.8
Leptin (ng/mL)	5.27 ± 0.50	4.61 ± 0.24	4.41 ± 0.17

Data are means ± SE; *n* = 10–12 animals from four litters. Where two or three pups from one litter were studied, their data were averaged. Different letters indicate significant differences between the groups (*P* < 0.05).

**Table 2:** Plasma analytes in fasted 91-day-old progeny from fructose- or glucose-supplemented mothers.

	Male		
	Control	Fructose	Glucose
Glucose (mg/dL)	89.3 ± 6.1	97.6 ± 4.8	96.8 ± 5.7
Insulin (*μ*g/L)	0.044 ± 0.015^a^	0.144 ± 0.031^b^	0.031 ± 0.006^a^
Triglycerides (mg/dL)	52.1 ± 12.6	53.8 ± 6.8	35.6 ± 4.1
NEFA (mM)	1.66 ± 0.24	1.37 ± 0.13	1.42 ± 0.06
Glycerol (mg/dL)	5.04 ± 0.72	4.12 ± 0.34	4.25 ± 0.15
Adiponectin (*μ*g/mL)	29.9 ± 6.8	27.7 ± 3.6	21.4 ± 1.0
Ketone bodies (mM)	0.64 ± 0.16	0.63 ± 0.11	0.73 ± 0.06

	Female		
	Control	Fructose	Glucose

Glucose (mg/dL)	104.1 ± 6.6	94.8 ± 2.8	102.9 ± 7.4
Insulin (*μ*g/L)	0.101 ± 0.017^a^	0.042 ± 0.010^b^	0.026 ± 0.004^b^
Triglycerides (mg/dL)	26.4 ± 8.9	24.7 ± 4.4	23.6 ± 4.6
NEFA (mM)	1.49 ± 0.29	1.49 ± 0.21	1.29 ± 0.04
Glycerol (mg/dL)	4.62 ± 0.43	4.32 ± 0.48	4.94 ± 0.58
Adiponectin (*μ*g/mL)	47.8 ± 8.6	48.2 ± 2.0	37.6 ± 5.3
Ketone bodies (mM)	0.63 ± 0.10	0.97 ± 0.12	1.00 ± 0.20

Data are means ± SE; *n* = 10–12 animals from four litters. Where two or three pups from one litter were studied, their data were averaged. Different letters indicate significant differences between the groups (*P* < 0.05).
